# Mitochondrial K_ATP_ channels contribute to the protective effects of hydrogen sulfide against impairment of central chemoreception of rat offspring exposed to maternal cigarette smoke

**DOI:** 10.1371/journal.pone.0237643

**Published:** 2020-10-16

**Authors:** Fang Lei, Wen Wang, Yating Fu, Ji Wang, Yu Zheng

**Affiliations:** 1 West China Hospital, Sichuan University, Chengdu, Sichuan, P.R. China; 2 Department of Physiology, West China School of Basic Medical Sciences and Forensic Medicine, Sichuan University, Chengdu, Sichuan, P.R. China; Virginia Commonwealth University, UNITED STATES

## Abstract

We previously reported that maternal cigarette smoke (CS) exposure resulted in impairment of central chemoreception and induced mitochondrial dysfunction in offspring parafacial respiratory group (pFRG), the kernel for mammalian central chemoreception. We also found that hydrogen sulfide (H_2_S) could attenuate maternal CS exposure-induced impairment of central chemoreception in the rat offspring *in vivo*. Mitochondrial ATP sensitive potassium (mitoK_ATP_) channel has been reported to play a significant role in mitochondrial functions and protect against apoptosis in neurons. Thus, we hypothesize here that mitoK_ATP_ channel plays a role in the protective effects of H_2_S on neonatal central chemoreception in maternal CS-exposed rats. Our findings revealed that pretreatment with NaHS (donor of H_2_S, 22.4mM) reversed the central chemosensitivity decreased by maternal CS exposure, and also inhibited cell apoptosis in offspring pFRG, however, 5-HD (blocker of mitoK_ATP_ channels, 19mM) attenuated the protective effects of NaHS. In addition, NaHS declined pro-apoptotic proteins related to mitochondrial pathway apoptosis in CS rat offspring pFRG, such as Bax, Cytochrome C, caspase9 and caspase3. NaHS or 5-HD alone had no significant effect on above indexes. These results suggest that mitoK_ATP_ channels play an important role in the protective effect of H_2_S against impairment of central chemoreception via anti-apoptosis in pFRG of rat offspring exposed to maternal CS.

## Introduction

Central chemoreflex is important for regulation of mammalian rhythmic respiratory activity, in which parafacial respiratory group (pFRG) is considered as an important central chemoreceptor [1,2]. Abnormal prenatal development of pFRG is involved in sudden infant death syndrome and congenital hypoventilation syndrome, whose victims suffer from central chemoreflex reduction and central apnea [3,4]. Maternal cigarette smoke (CS) exposure during pregnancy greatly increases the risk of sudden infant death syndrome [5,6]. We have found that maternal CS exposure leads to a deficit in central chemoreception of neonatal rats [7]. However, the underlying mechanism is not yet being completely understood.

CS contains a large amount of toxic agents, including nicotine, carbon monoxide, heavy metals, as well as reactive oxygen species (ROS) [8]. We previously found that maternal CS exposure increased oxidative stress and mitochondrial dysfunction in offspring pFRG in rat model [9]. It has been shown that excessive ROS production can induce mitochondrial pathway of apoptosis by bringing damages to mitochondria, including mitochondrial DNA, proteins, and lipids [10]. Thus, we propose that maternal CS exposure may result in cell apoptosis in offspring pFRG, which might be involved in the impairment of central chemoreception in the offspring.

Hydrogen sulfide (H_2_S), as a signaling molecule, can be endogenously generated by cystathionine β-synthase, cystathionine γ-lyase and 3-mercaptopyruvate sulfurtransferase [11]. It has been reported to serve as an important modulator and protectant in central nervous system [12]. For instance, H_2_S regulates calcium homeostasis in microglia and neurons [13,14]. H_2_S also enhances NMDA receptor-mediated excitatory postsynaptic potential and facilitates the induction of long-term potentiation [15]. In addition, studies have clarified the potent protective roles of H_2_S in several central nervous system diseases. For example, H_2_S can protect neurons from oxidative stress in AD [16], PD [17] and traumatic brain injury [18]. Furthermore, H_2_S can also protect neural cells from apoptosis by inhibiting mitochondrial apoptotic pathway [19]. Our previous study has suggested that H_2_S can protect neonatal central chemoreception against impairment resulting from maternal CS exposure *in vivo* [20]. However, the mechanism still remains unclear.

ATP sensitive potassium (K_ATP_) channels can be found in the plasma membrane and the inner membrane of mitochondria [21]. The mitochondrial K_ATP_ (mitoK_ATP_) channel was first identified by single channel patch-clamp recordings of rat liver mitochondrial inner membrane [22]. Accumulating evidence has demonstrated that mitoK_ATP_ channel is important for protecting myocardial cells against injuries [23,24]. It has been shown that mitochondria within the brain contain much more abundant mitoK_ATP_ channels than cardiac tissues [25], which suggests an important role of the channels in the brain. Studies have indicated that mitoK_ATP_ channels agonist can inhibit apoptosis induced by hydrogen peroxide [26]. MitoK_ATP_ channels are also suggested to mediate neuroprotection induced by chronic morphine preconditioning in hippocampal CA-1 neurons following cerebral ischemia [27]. Here, we sought to investigate whether mitoK_ATP_ channels are involved in the protective effect of H_2_S on the central chemoreception of neonatal rats exposed to maternal CS.

## Materials and methods

The experimental protocols were approved by the Animal Care and Use Committee of Sichuan University, and all studies were performed in accordance with the national institute of health guide for the care and use of laboratory animals (NIH publication No.8023) revised 1978.

### Treatment of animals

Adult Sprague Dawley rats were obtained from Sichuan University experimental animal center. At the beginning of experiment, rats were kept in a temperature-controlled (~25°C) room with a 12h light/dark cycle and had free access to food and water. Pregnancy was confirmed by the presence of spermatozoa on the vaginal smear and the following day was considered as gestational day (gd) 1. The corresponding 2-day-old pups were used in the studies, and randomization was performed within each litter in all experimental groups.

Pregnant rats were divided into six groups: Control, CS, CS+NaHS (donor of H_2_S), CS+NaHS+5-HD (a blocker of mitoK_ATP_ channels), NaHS, 5-HD. To mimic active smoking during pregnancy as closely as possible, maternal CS exposure was designed based on the model we used before with a modification [7,28]. Briefly, the exposure duration was changed to gd1-20 from gd7-20. Daily CS exposure was performed in CS, CS+NaHS and CS+NaHS+5-HD groups in two sessions, one in the morning starting at 9:00 and one in the afternoon starting at 16:00. For each session, pregnant rats were placed in a restraining exposure box and distributed to CS cyclically (2 cigarettes (Tianxiaxiu, 11 mg of tar and 1 mg of nicotine per cigarette, China Tobacco Chuanyu Industrial Co., China)/12 min, 10 min with the box closed and the remaining 2 min with the box open, five times for one session). With this regimen, serum cotinine concentration (92.3±15.7 ng/ml) [28] achieves a level of smoking exposure that simulates active smoking during pregnancy [29,30]. At the same time, a similar procedure was used for animals in the Control, NaHS and 5-HD groups except that they were exposed to room air rather than to CS in an identically sized exposure box.

In the present study, NaHS and 5-HD (Sigma, German) were dissolved in physiological saline and prepared freshly before daily injection during gd1-20. Pregnant rats received intraperitoneal injection of NaHS solution (22.4mM, 2.5ml/kg body weight) 30 min before the first session of daily exposures in the CS+NaHS, NaHS and CS+NaHS+5-HD groups. Pregnant rats in the Control and CS groups received injections of equivalent volume of physiological saline. Pregnant rats in CS+NaHS+5-HD and 5-HD groups received intraperitoneal administration of 5-HD solution (19mM, 2.5ml/kg body weight) 60min before CS or room air exposures.

None of the pregnant rats became severely ill or moribund during the experiment, therefore all animals survived until the experimental endpoint. The dams were humanely euthanized after giving birth with sodium pentobarbital by intraperitoneal injection.

### Assessment of central chemoreception in medullary preparations

Transverse medullary preparations were prepared from neonatal rats as previously described [7,28]. In brief, the neonates were anesthetized with ether inhalation and then were decapitated. Brainstems were isolated in ice-cold oxygenated (95% O_2_-5% CO_2_) artificial cerebrospinal fluid (ACSF, pH 7.4), composed of (in mM): 125 NaCl, 3 KCl, 1.2 CaCl_2_, 1 MgSO_4_, 22 NaHCO_3_, 1 NaH_2_PO_4_ and 30 D-glucose. The brainstem was glued to an agar block with superglue, caudal surface up, for micro-sectioning in a vibrating microtome (Campden Instrument LTD, UK). A single medullary preparation, corresponding to the level approximately from the obex to 1800μm rostral to the obex, was taken to capture the preBötinger complex and pFRG, kernels for the generation of respiratory rhythm and central chemoreception, respectively. The preparation was transferred to fresh standard ACSF that was continuously oxygenated with 95% O_2_-5% CO_2_, and incubated for about 60min at 28–29°C. The preparation was then transferred to the recording chamber which was continuously perfused (4ml/min) with standard ACSF. During experiments, rhythmic respiratory activity of hypoglossal rootlets was maintained by elevating the superfusate K^+^ concentration to 8mM.

The rhythmic respiratory-like discharges of hypoglossal rootlets were recorded by using glass suction electrodes filled with ACSF. Signals were amplified, filtered (τ = 0.001s, F = 1 kHz) and integrated (time constant of 50ms) by using a BL-420F Biological Signal Processing System (Taimeng Biotech. Co., China). Discharges of hypoglossal rootlets of the medullary preparations in the standard ACSF for 5min were recorded as the baseline after the discharges were stable. Acidified ACSF (pH 7.0) was then applied to preparations for another 5min and then a 20-min period for washout. pH of the ACSF was obtained by adding HCl (5mM) or NaOH (5mM). Burst frequency (BF) changes of hypoglossal rootlets were analyzed to reflect the central chemoreception of the rat pups.

### pFRG sample collection

At postnatal day 2, rat pups were sacrificed by decapitation after being anesthetized with ether inhalation. Brainstems were isolated, and then 900μm-thick medullary slices (corresponding to the level of the medulla from approximately 900μm to 1800μm rostral to the obex) containing pFRG were obtained by a vibrating microtome (Campden Instrument LTD, UK), and pFRG was sampled from the slices and stored at −80°C for further detection.

### Terminal deoxynucleotidyl transferase dUTP nick end labeling assay

Terminal deoxynucleotidyl transferase dUTP nick end labeling (TUNEL) assay was used to determine the apoptosis of pFRG cells with an *in situ* cell death detection kit (Roche, Switzerland) according to the manufacturer’s instruction. Brainstems of neonatal rats were dissected and immersed in 4% paraformaldehyde overnight at 4°C. Then the specimens were processed and embedded in paraffin. Transverse sections (5μm) containing pFRG were obtained. After dewaxing and rehydration, tissue sections were incubated in proteinase K working solution (20μg/ml) at 37°C for 15min. Then, sample slides were rinsed twice with PBS. Followed by drying area around samples, 50μl TUNEL reaction mixture (50μl enzyme solution: 450μl label solution) was added on samples. For negative control, only 50μl label solution was added. For positive control, prior to labeling, sections were incubated with DNase to induce DNA strand breaks. All sections were incubated at 37°C for 60min in a humidified atmosphere in the dark, and then slides were rinsed with PBS for 3 times. Nuclei were counterstained with hematoxylin. The apoptotic index was calculated as the ratio of apoptotic cell number to total cell number.

### Examination of enzyme activities of caspase3 and caspase9

Enzyme activities of caspase3 and caspase9 were tested with corresponding assay kits (KeyGENBioTECH, China) according to the manufacturer’s instruction. Briefly, pFRG supernatant containing 200μg protein was added to the reaction reagents for caspase3 and caspase9 activity assays, respectively, and the mixture was incubated for 4h at 37°C in dark. The absorbance was measured at 405nm using a microplate reader (Thermo Fisher, USA).

### Western blotting analysis

pFRG tissues were homogenized in radio immunoprecipitation assay buffer with phenylmethanesulfonyl fluoride protease. The homogenate was centrifuged at 4°C, 12,000r/min for 15min. Protein concentration of samples was determined by using BCA protein assay kit. Then, proteins (30μg/lane) were electrophoresed by SDS-PAGE and transferred to PVDF membranes. After blocked in 5% skim milk for 2h at room temperature, the membranes were subsequently incubated with primary antibodies overnight at 4°C, and then with second antibodies for 90min at room temperature. The chemiluminescence results were recorded by an imaging system (V140130, Bio-Rad, USA). Signal intensities were measured using Image Lab software (Bio-Rad, USA). Antibodies used in the present study were as follows: rabbit anti-Bax (1:1000, Santa Cruz, USA), rabbit anti-Bcl-2 (1:400, Proteintech, USA), rabbit anti-Apaf-1 (1:400, Proteintech, USA), mouse anti-CytC (1:3000, Ruiying Biological, China), rabbit anti-caspase3 (1:800, Cell Signaling Technology, USA), rabbit anti-caspase9 (1:500, Santa Cruz, USA), mouse anti-GAPDH (1:4000, Servicebio, China), rabbit anti-β-actin (1:2000, Bioss, China).

### Statistical analysis

Statistical analysis was performed using repeated measures ANOVA for results of fictive respiratory activity recordings, and two-way ANOVA for other results. All data were presented as mean ± SEM. Statistical significance was set at *P*<0.05.

## Results

### MitoK_ATP_ channel is involved in the protective effect of H_2_S against impairment of central chemoreception of the offspring

**Fig 1:**
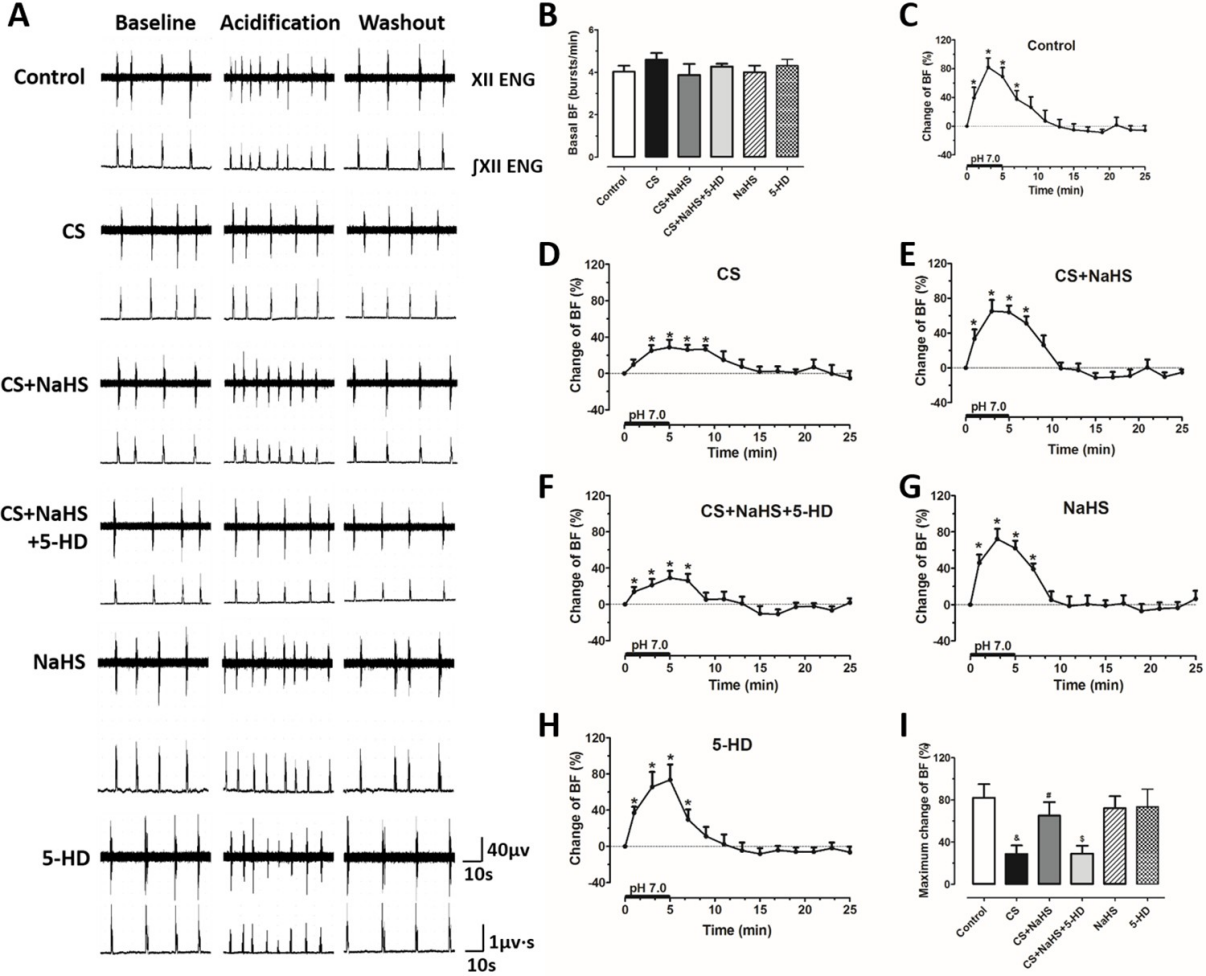
MitoK_ATP_ channel blocker restrains the protective effect of NaHS on maternal CS exposure-induced impairment of central chemoreception in offspring medullary slices. (A) Typical recordings of hypoglossal rootlets discharges in medullary slices of the offspring. In each panel, the upper and lower lines indicate the raw (XII ENG) and integrated (∫XII ENG) activities of hypoglossal rootlets during baseline, acidification and washout, respectively; (B) comparison of basal burst frequency (BF) in different groups. Changes of BF in the (C) Control group (n = 8), (D) CS group (n = 8), (E) CS+NaHS group (n = 8), (F) CS+NaHS+5-HD group (n = 6), (G) NaHS group (n = 8) and (H) 5-HD group (n = 6); (I) comparison of maximum changes of BF of hypoglossal rootlets discharges responding to acidified perfusion among all groups. XII ENG: electroneurogram of hypoglossal rootlets, ∫XII ENG: integrated XII ENG. *P<0.05 vs. 0 min; ^&^*P*<0.05 vs. Control group; ^#^*P*<0.05 vs. CS group; ^$^*P*<0.05 vs. CS+NaHS group.

We have known that maternal CS exposure inhibits the ventilatory response to hypercapnia of neonatal rats, which can be reversed by H_2_S [20]. Here, we found that when the preparations were perfused with standard ACSF, the basal BF of hypoglossal rootlets was not significantly different among groups (*P*>0.05, Fig 1A and 1B); under the perfusion of acidified ACSF, BF was obviously increased in all groups (*P*<0.05, Fig 1A and 1C–1H). We then compared the incremental quantity among groups, and we found that the increase in BF induced by acidification in preparations from maternal CS-exposed rats was visibly smaller than that from Control rats (*P*<0.05, Fig 1I), which was in line with what we observed before [7]; NaHS reversed maternal CS exposure-induced reduction in BF increase responding to acidification (*P*<0.05, Fig 1I); however, in the CS+NaHS+5-HD group, BF increase was markedly inhibited as compared with that in the CS+NaHS group (*P*<0.05, Fig 1I). These results indicate that H_2_S can attenuate maternal CS exposure-induced impairment of central chemoreception of the offspring, whereas this effect can be hindered by mitoK_ATP_ blocker.

### MitoK_ATP_ channel is involved in the protective effect of H_2_S against apoptosis in offspring pFRG

**Fig 2:**
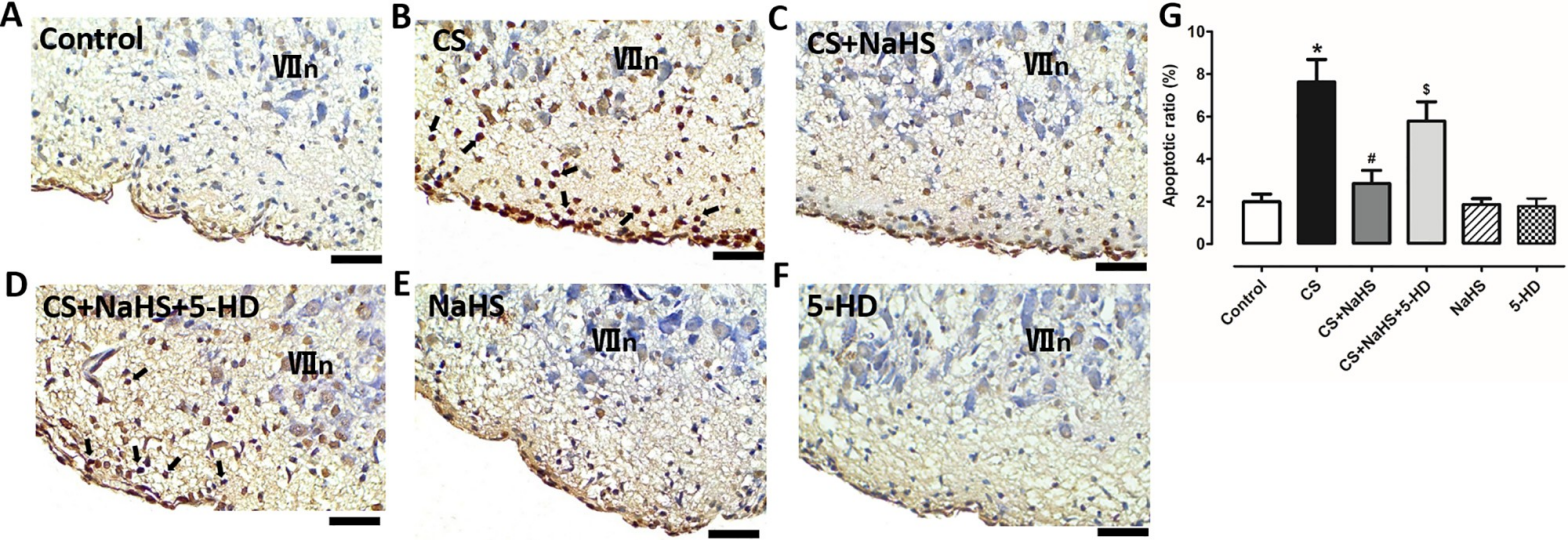
MitoK_ATP_ channel blocker inhibits the protective effect of NaHS on maternal CS exposure-induced cell apoptosis in offspring pFRG. Representative images of cellular TUNEL staining in pFRG of neonatal rats from (A) Control group, (B) CS group, (C) CS+NaHS group, (D) CS+NaHS+5-HD group, (E) NaHS group, and (F) 5-HD group; (G) comparison of apoptotic ratios in pFRG of neonatal rats among all groups. *P<0.05 vs. Control group; ^#^*P*<0.05 vs. CS group; ^$^*P*<0.05 vs. CS+NaHS. n = 5. Black arrows show representative apoptotic cells. Scale bars represent 20μm. VIIn: facial nucleus.

As pFRG is the key chemoreceptor for central chemoreception of the mammals, injuries in pFRG will lead to insufficiency of central chemosensitivity. Thus, in the present study, we examined cell apoptosis in offspring pFRG. We first detected the apoptosis in offspring pFRG directly by TUNEL staining. As shown in Fig 2, maternal CS exposure induced condensed nuclei, a characteristic of apoptosis, and the apoptotic ratio was significantly increased in comparison with that in the Control group (*P*<0.05); NaHS significantly attenuated such effect of maternal CS exposure (*P*<0.05). However, intraperitoneal injection of 5-HD significantly attenuated the inhibitory effect of NaHS on maternal CS exposure-induced cell apoptosis in pFRG (*P*<0.05) of the offspring; NaHS and 5-HD themselves had no significant effect on apoptosis (*P*>0.05).

**Fig 3:**
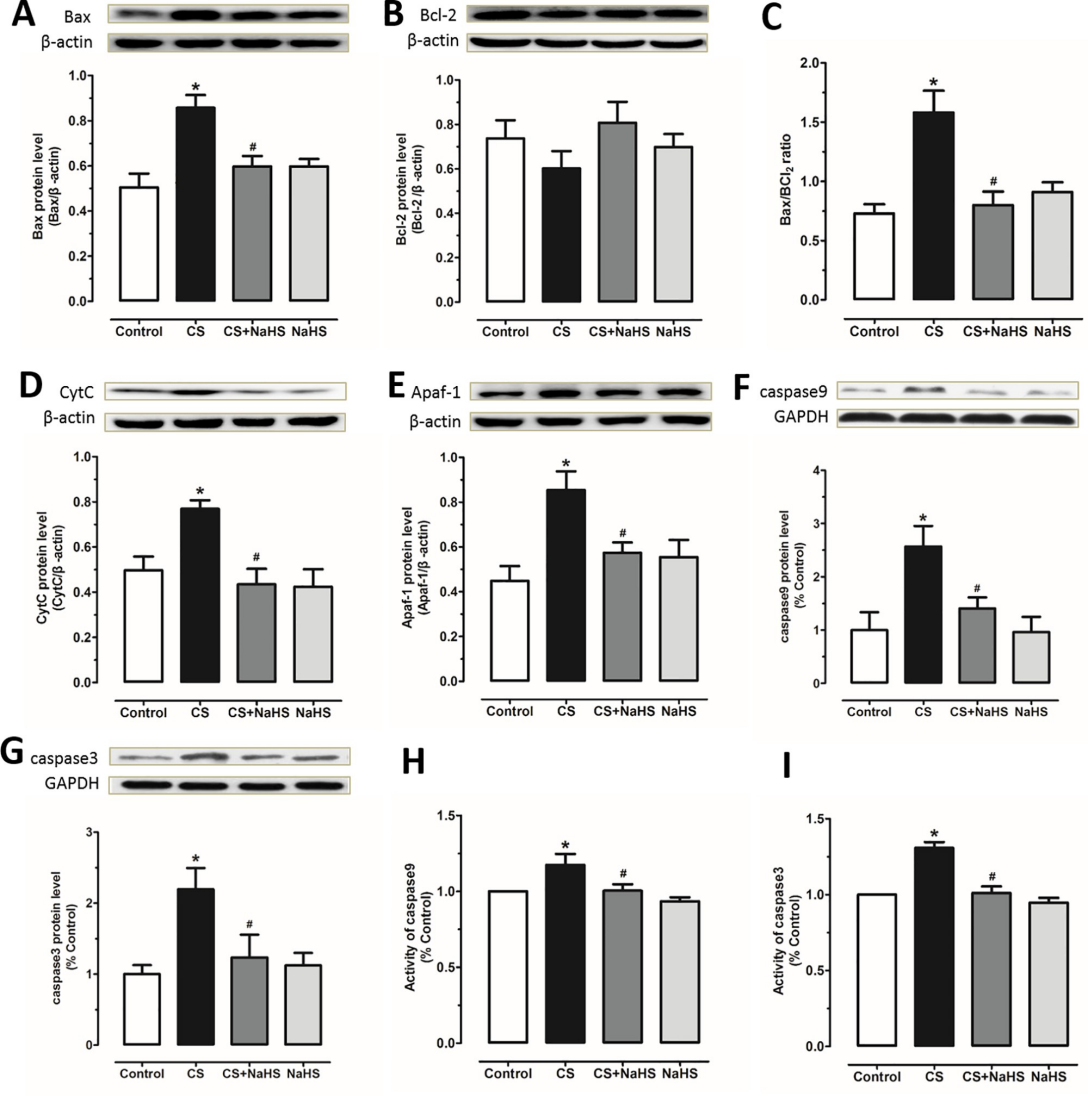
NaHS reverses maternal CS exposure-induced changes of mitochondrial apoptosis-related protein levels in offspring pFRG. Comparisons of protein expressions of (A) Bax (n = 5), (B) Bcl-2 (n = 5), (C) Bax/Bcl-2 ratio, protein expressions of (D) CytC (n = 6), (E) Apaf-1 (n = 5), (F) caspase9 (n = 5) and (G) caspase3 (n = 3), and enzyme activities of (H) caspase9 (n = 6) and (I) caspase3 (n = 6) in pFRG of neonatal rats from all groups. *P<0.05 vs. Control group; ^#^*P*<0.05 vs. CS group.

Then, proteins related to mitochondrial pathway of apoptosis were measured. Bax is a pro-apoptotic protein, whereas Bcl-2 is a negative regulator of apoptosis [31]. We found that Bax expression was increased in pFRG of offspring exposed to maternal CS compared with that in the Control group; NaHS reversed the effect of maternal CS exposure (*P*<0.05, Fig 3A). However, no change was observed in Bcl-2 protein level among groups (*P*>0.05, Fig 3B). Then, the ratio of Bax/Bcl-2 was compared. It was shown that the ratio was increased in the CS group and NaHS inhibited this increase (*P*<0.05, Fig 3C).

Increased Bax promotes CytC release from mitochondria, and after releasing, CytC will combine with apoptotic protease activating factor-1 (Apaf-1), and then activate the cascade reactions [31]. Next, we measured expressions of CytC and Apaf-1. Our data showed that maternal CS exposure augmented protein levels of both CytC and Apaf-1, which were suppressed by NaHS (*P*<0.05, Fig 3D and 3E).

CytC combines with Apaf-1 and pro-caspase9 to form apoptosome, which may activate caspase9 and then caspase3. Caspase3 is a crucial executioner of apoptosis [31]. In the present study, it was found that maternal CS exposure induced significant increases in caspase9 (*P*<0.05, Fig 3F) and caspase3 (*P*<0.05, Fig 3G) expressions. In addition, enzyme activities of caspase9 (*P*<0.05, Fig 3H) and caspase3 (*P*<0.05, Fig 3I) were also increased in CS group compared with the Control group. These effects of maternal CS exposure were markedly attenuated by NaHS administration (*P*<0.05, Fig 3F–3I).

## Discussion

Maternal CS or nicotine exposure has been reported to blunt central chemoreception in the offspring [7,20,32]. In the present study, we demonstrated that NaHS, a donor of H_2_S, was able to ameliorate the inhibitory effect of maternal CS exposure on neonatal central chemoreception *in vitro*, which is in line with what we have observed *in vivo* [20]. The underlying mechanisms might be associated with the mitoK_ATP_ channels-mediated apoptosis in the offspring pFRG.

We have reported that maternal CS exposure resulted in excessive ROS generation and impaired mitochondrial functions in offspring pFRG including reduced MMP, decreased mitochondrial DNA copy number and declined ATP production [9]. We also found that application of H_2_S inhibited maternal CS exposure-induced ROS overproduction and protected mitochondrial functions (data unpublished). MitoK_ATP_ channels contribute to the mitochondrial structure and function maintenance. Though numerous studies have indicated that mitoK_ATP_ channel activation is an important protective mechanism of cardiovascular injuries [33–36], mitoK_ATP_ channel is also reported to be involved in the pathogenesis of neurodegenerative diseases [37–39]. Studies have shown that mitoK_ATP_ channels are involved in the preservation of mitochondrial function [40]. Opening of mitoK_ATP_ channels leads to mitochondrial depolarization which is proposed to reduce mitochondrial Ca^2+^ uptake, thereby preventing Ca^2+^ overload [41,42], and opening of mitochondrial permeability transition pore that is known to facilitate the release of pro-apoptotic proteins [43].

It has been shown that mitoK_ATP_ channel activator can inhibit apoptosis which would be blocked by 5-HD [44,45]. K_ATP_ channels are considered primary molecular targets for H_2_S [46]. It is reported that 4-carboxyphenyl isothiocyanate, a donor of H_2_S, significantly improved the recovery of myocardial function, and pre-treatment with 5-HD abolished the protective effect of H_2_S [47]. In the present study, we examined the role of mitoK_ATP_ channel in the protective effect of H_2_S on the central chemoreception of CS-rat offspring. We measured the levels of proteins related to mitochondrial pathway of apoptosis. Bax and Bcl-2 are members of Bcl-2 family proteins, and they play important roles in regulation of mitochondrial pathway of apoptosis. Apoptotic signals promote Bax, a pro-apoptotic protein, to translocate to the outer mitochondrial membrane to increase mitochondrial membrane permeability, which induces CytC release from mitochondria, leading to mitochondrial pathway of apoptosis. Bcl-2, an anti-apoptotic protein, prevents this process [48]. We found that maternal CS exposure increased Bax expression and augmented the ratio of Bax/Bcl-2, and it also increased CytC protein level. CytC released from mitochondria forms a complex with Apaf-1 and then activates caspase9, which may activate caspase3, the executioner of apoptosis [31]. Indeed, in the present study, maternal CS exposure not only elevated protein levels of caspase9 and caspase3, but also increased their activities, and finally induced mitochondrial pathway of apoptosis in offspring pFRG. H_2_S could protect the offspring pFRG from injury of apoptosis, which was beneficial for the recovery of central chemoreception. However, these protective effects of H_2_S were blocked by 5-HD.

It has been reported that mitoK_ATP_ channels are associated with the maintenance of mitochondrial matrix volume and block of proapoptotic proteins release [40,49]. Although we did not directly prove that mitoK_ATP_ channels have a role in the inhibiting effect of H_2_S on maternal CS exposure-induced pro-apoptotic protein release, in our previous study, we found that maternal CS exposure resulted in increased ROS generation, mitochondrial swelling, loss of mitochondrial membrane potential (MMP) and decreased ATP production [9], furthermore, in this study, when H_2_S (a potassium channel opener) was given, the apoptosis was significantly attenuated, but 5-HD (a mitoK_ATP_ channel inhibitor) reversed the protective effect of H_2_S. These results together indicate that mitoK_ATP_ channels are involved in the inhibition of pro-apoptotic proteins release by H_2_S.

In addition to mitoK_ATP_ channels, kinases are also reported to be involved in the regulation of apoptosis by H_2_S. Studies have indicated that H_2_S can reduce apoptosis through ROS/MAPK pathway [50–52]. ROS can activate MAPKs, and apoptotic cell death induced by ROS can be mediated by the MAPK pathway. H_2_S inhibits ROS generation, increases the activity of antioxidant enzyme, suppresses phosphorylations of MAPKs and decreases apoptosis. It was also showed that the activation of PI3K/Akt signaling was involved in the anti-apoptosis effect of H_2_S [53–55]. Thus, further study is still needed to explore the detailed mechanism of maternal CS exposure-induced impairment of neonatal central chemoreception and the protective effect of H_2_S.

The present study provides evidence that mitoK_ATP_ channels are related to apoptosis in pFRG that is associated with maternal CS exposure-induced impairment of neonatal central chemoreception, and H_2_S can protect offspring pFRG from apoptosis via the activation of mitoK_ATP_ channels, which contributes to the recovery of central chemoreception. Thus, our data suggest that H_2_S may have potential therapeutic value for maternal CS exposure induced central chemoreception deficit-related diseases, such as sudden infant death syndrome.

## Supporting information

S1 Raw images (PDF)
