# Supplementary material for: Mitochondrial KATP channels contribute to the protective effects of hydrogen sulfide against impairment of central chemoreception of rat offspring exposed to maternal cigarette smoke
Source: PLoS One. 2020 Oct 16;15(10):e0237643. doi: 10.1371/journal.pone.0237643 (PMC7567348; doi:10.1371/journal.pone.0237643)
Supplement: S1 Raw images — (PDF) [file pone.0237643.s001.pdf]

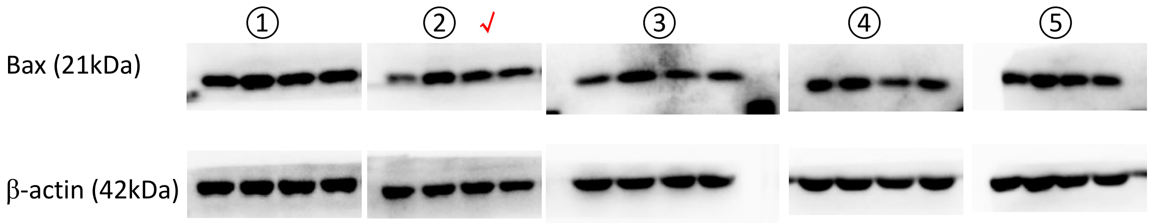

The loading order for Bax protein: Control, CS, CS+NaHS and NaHS.

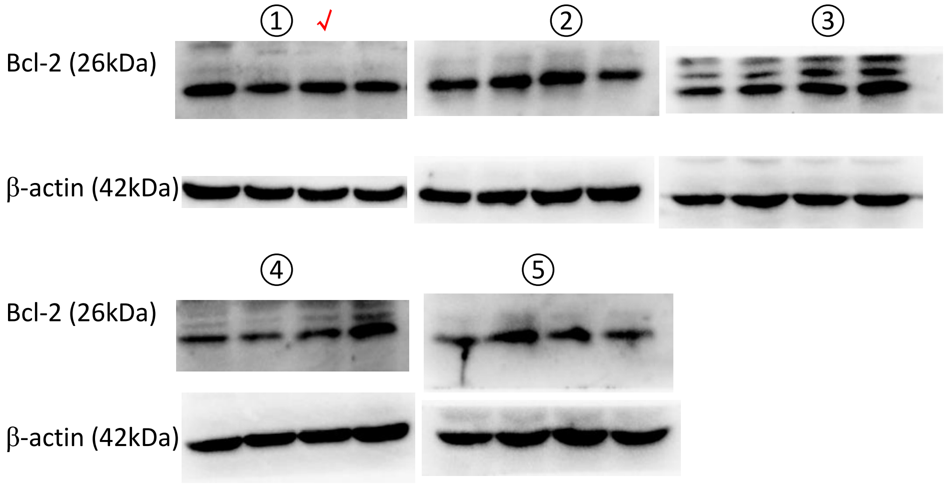

The loading order for Bcl-2 protein: Control, CS, CS+NaHS and NaHS.

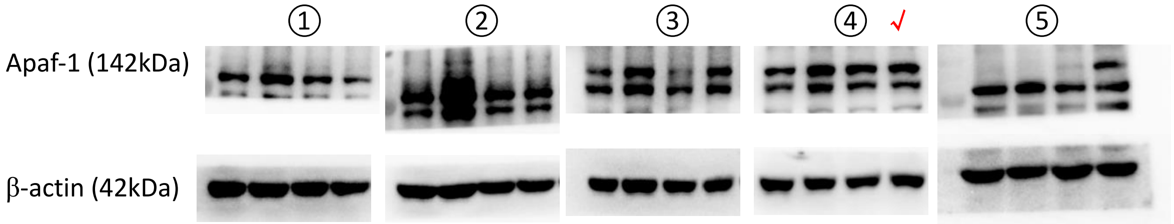

The loading order for Apaf-1 protein: Control, CS, CS+NaHS and NaHS.

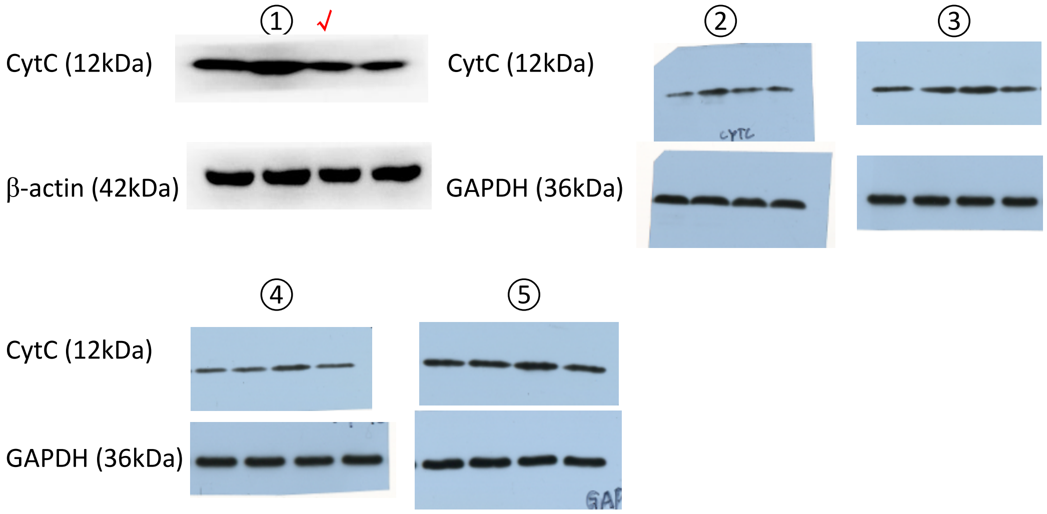

The loading order for CytC protein in panel ①-②: Control, CS, CS+NaHS and NaHS; in panel ③-⑤: Control, NaHS, CS and CS+NaHS.

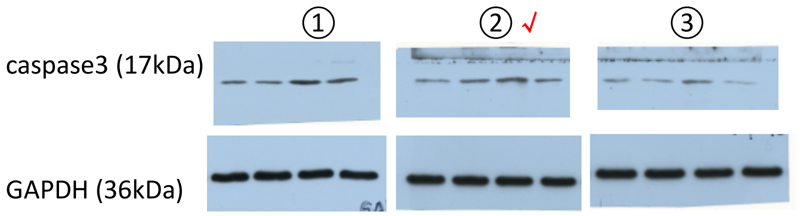

The loading order for caspase3 protein: Control, NaHS, CS and CS+NaHS.

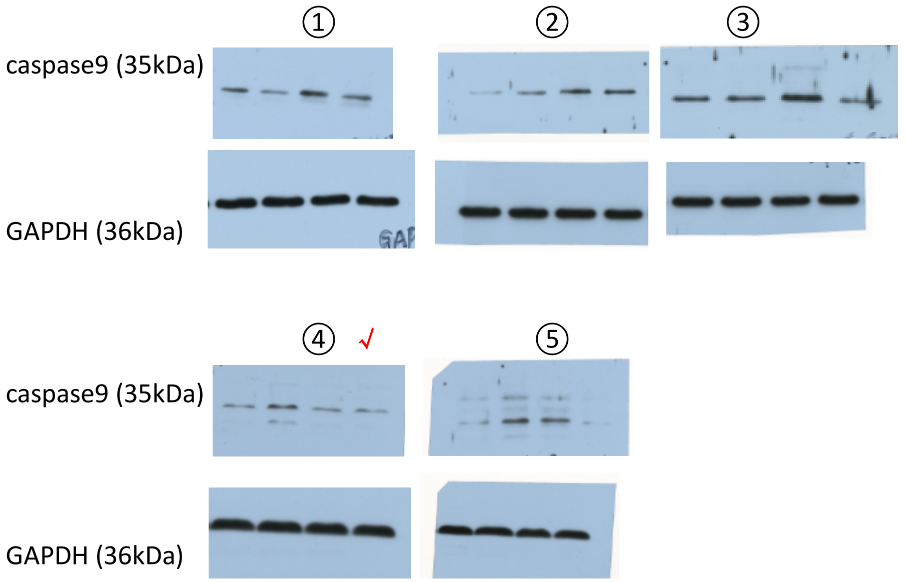

The loading order for caspase9 protein in panel ①-③: Control, NaHS, CS and CS+NaHS; in panel ④-⑤: Control, CS, CS+NaHS and NaHS.

The original images with " √ " were used in the Fig 3 for corresponding protein.
